# Depressive and anxiety symptoms, and neural correlates of reward and punishment anticipation in female athletes with amenorrhea

**DOI:** 10.3389/fendo.2023.976050

**Published:** 2023-05-18

**Authors:** Charumathi Baskaran, Poornima Kumar, Franziska Plessow, Supritha Nimmala, Kathryn E. Ackerman, Kamryn T. Eddy, Diego A. Pizzagalli, Madhusmita Misra

**Affiliations:** ^1^ Department of Pediatrics, Boston Children’s Hospital and Harvard Medical School, Boston, MA, United States; ^2^ Neuroendocrine Unit, Massachusetts General Hospital and Harvard Medical School, Boston, MA, United States; ^3^ Center for Depression, Anxiety and Stress Research, McLean Hospital, Belmont, MA, United States; ^4^ Department of Psychiatry, Boston Children’s Hospital and Harvard Medical School, Boston, MA, United States; ^5^ Department of Sports Medicine, Harvard Medical School, Boston, MA, United States; ^6^ Eating Disorders Clinical and Research Program, Massachusetts General Hospital, Boston, MA, United States; ^7^ Division of Pediatric Endocrinology, Massachusetts General Hospital for Children and Harvard Medical School, Boston, MA, United States

**Keywords:** estradiol, adolescent, anxiety, depression, reward and punishment processing

## Abstract

**Objective:**

Studies in estrogen deficiency states such as primary ovarian insufficiency and Turner syndrome suggest that estrogen status may be an important modulator of mood and emotions. In this study we compared depressive and anxiety symptoms between adolescent and young adult female oligo-amenorrheic athletes (AA) and eumenorrheic females (EM), and explored structural, and functional changes in related brain areas during reward processing, a behavioral construct that is altered in depression and anxiety.

**Methods:**

We included (i) 24 AA participating in ≥4 hours/week of aerobic exercise or running ≥20 miles/week for ≥6 months in the preceding year, with lack of menstrual cycles for ≥3 months within at least 6 preceding months of oligo-amenorrhea, OR in premenarchal girls, absence of menses at >15 years), and (ii) 27 EM aged 14-25 years. Participants completed the Beck Depression Inventory-II (BDI-II), State and Trait Anxiety Inventory (STAI), and Mood and Anxiety Symptoms Questionnaire (MASQ). Structural MRI and brain activation during a functional MRI (fMRI) task that probes reward and punishment processing was examined in a subset of 10 AA and 23 EM.

**Results:**

Median (IQR) age and BMI of AA and EM groups were 20.6 (19.0-22.6) vs. 20.6 (19.2-23.7) years, p=0.6 and v 20.3 (18.8-21.5) vs. 21.9 (19.6-23.5) kg/m2, p=0.005, respectively. While groups did not differ for BDI-II scores, AA had higher anhedonic depression MASQ scores (p=0.04), and STAI (p=0.03) scores vs. EM. In the fMRI subset, AA had higher caudate volumes vs. EM [F(1, 29)=9.930, p=0.004]. Lower activation observed in the right caudate during reward anticipation in AA compared with EM (p=0.036) suggests blunted reward processing in the striatum in estrogen deficient states.

**Conclusion:**

Athletes with amenorrhea had higher depressive and anxiety symptomatology compared to eumenorrheic young women. Exploratory analyses demonstrated increased caudate volumes and decreased caudate activation during reward processing in athletes with amenorrhea suggesting that estrogen may play a role in reward processing.

## 1 Introduction

Approximately 11.3% of adolescents in the United States between ages 12-17 years have one episode of major depression in the past year (1) and about 31.9% develop anxiety disorders between the ages of 13-18 years with a higher prevalence in females (2). These disorders are a major cause of disability among adolescents and young adults, and a significant risk factor for suicide, which is the third leading cause of death for adolescents aged 15-19 years (3). Understanding the factors that impact mood and anxiety in adolescents may provide novel direction regarding the management of these disorders.

Emerging evidence indicates that estrogen may play a significant role in modulating mood. In particular, estrogen deficiency states such as menopause and Turner syndrome have been associated with a higher risk of depression and anxiety compared to the general population (4, 5). Exercise without appropriate nutritional fueling can result in low energy availability, even in normal-weight athletes; this disrupts the hypothalamic-pituitary-gonadal (HPG) axis, leading to functional hypothalamic amenorrhea (FHA) and low estrogen levels (6). While numerous reports have demonstrated a positive effect of exercise on depression and anxiety (7, 8), the role of estrogen deficiency resulting from physical activity in young athletes with amenorrhea on mood and anxiety has not been studied in detail. To our knowledge, only one study has evaluated mood issues in athletes with respect to their menstrual status and reported a trend for a higher prevalence of bipolar disorders or major depressive disorders in 23% of runners who had absent menses (amenorrhea) versus 0% runners with regular menses (eumenorrhea) (9). Similarly, an increased prevalence of depressed mood has been reported in FHA similar to that in organic amenorrhea, suggesting that estrogen deficiency might be a common factor in both groups driving mood issues (10, 11). Of relevance, we recently reported improvement of trait anxiety with estrogen replacement in girls with FHA (12). However, studies evaluating depression and anxiety in athletes who have estrogen deficiency and are amenorrheic are limited.

One of the hallmarks of mood disorders is abnormal reward and punishment processing that is often associated with structural and functional changes in reward-related brain regions, most prominently the striatum, including the caudate, putamen and the nucleus accumbens (NAc) (13–15). Of note, the ability to anticipate reward is often blunted in depression and is a core behavioral construct that is frequently tested in functional neuroimaging studies examining depression. In general, these studies have shown reduced brain activation in the caudate while anticipating and receiving a reward among adolescents with depression (16). The anticipatory response to punishment is less well known in depression. However, induced anxiety has been reported to increase striatal activation to both rewards and punishment, suggesting anxiety symptoms may impact incentive processing generally (17). Based on these findings we designed a study to compare depressive and anxiety symptoms in adolescent and young adult athletes with irregular or absent menses (oligo-amenorrhea) versus athletes and non-athletes with regular menses (eumenorrhea). Further, we compared a subset of these subjects with respect to structural (volumetric) features of the striatum as well as functional striatal activation using a monetary incentive paradigm that probes a behavioral construct altered in depression/anxiety. We hypothesized that estrogen deficiency would negate the beneficial effects of exercise, and that compared to eumenorrheic females (EM), female athletes with oligo-amenorrhea (AA) would have greater symptoms of depression and anxiety. Additionally, we hypothesized that oligo-amenorrheic athletes would exhibit structural changes in the striatum (caudate, putamen and NAc) and have altered neural activation during reward/punishment anticipation when compared to the EM group. This novel study is the first of its kind to evaluate the relationship of menstrual status on mood in adolescent and young adult female athletes, and underlying neural correlates.

## 2 Methods

### 2.1 Participants

Study participants were recruited by advertising in institutional recruitment forums, sports medicine treatment centers and physician practices. The study included 51 right-handed females between ages 14-25 years with BMI between the 10th-90th percentiles: 24 AA, 14 EA and 13 NA. Participants were grouped by menstrual status into two categories: one with oligo-amenorrhea (AAs only, n=24) and the other with regular menses or eumenorrhea and no medical issues, EM (EA, and NA taken together, n=27). Athletes were defined as those participating in ≥4 hours/week of aerobic exercise or running ≥20 miles/week for a period of ≥6 months in the preceding year. NA did not participate in team sports and exercised <2 hours/week. Oligo-amenorrhea was defined as lack of menstrual periods for ≥3 months within at least 6 preceding months of oligo-amenorrhea, or in premenarchal girls, absence of menses at >15 years. Exclusion criteria included (a) conditions other than excessive exercise causing loss of periods, including pregnancy, polycystic ovary syndrome and other conditions of hyperandrogenism, thyroid dysfunction (unless euthyroid on medications for at least 3 months), primary ovarian insufficiency, and hyperprolactinemia, and (b) any contraindications to estrogen use such as an increased predisposition for thromboembolism, underlying liver disease, and a personal or first-degree family history of an estrogen dependent cancer. All participants (or their parents) provided written informed consent to the protocol, which was approved by our Institutional Review Board. Participants below 18 years also provided signed assent for study participation.

### 2.2 Procedure

Following a screening visit, participants completed questionnaires, had a structural MRI scan and completed a monetary incentive delay task in the scanner. Twenty-three AA and 26 participants from the eumenorrheic group, EM, completed questionnaires to assess their mood. Depressive, anxiety, and anhedonic symptoms were assessed using the Beck Depression Inventory-II (BDI-II) (18), the State-Trait Anxiety Inventory (STAI) (19, 20), and the Mood and Anxiety Symptoms Questionnaire (MASQ) (21) respectively. Imaging data were obtained in 10 AA and 23 EM participants. The majority of EM participants (except for 9) were brought in during the proliferative phase of their cycle, which was determined based on their last menstrual period. Estradiol levels were available for 9 AA and 23 EM participants. Estradiol was assessed by chemiluminescence (Beckman Coulter, Fullerton, CA; sensitivity 20 pg/mL; intraassay CV 2.0-4.2%). We used menstrual status (rather than an estradiol level) as a surrogate of estrogen status given that estradiol levels vary across the menstrual cycle and are dependent on cycle phase, and they do not necessarily reflect the chronicity of estrogen exposure. Further, standard estradiol assays are not always reliable at low levels.

### 2.3 MRI procedure

Please see the **Supplement** for detailed information on MRI procedures, data quality assurance, preprocessing, and statistical analysis.

### 2.4 Monetary incentive delay task

The monetary incentive delay task is designed to measure neural responses to anticipation and receipt of rewards and penalties and has been demonstrated to successfully recruit the striatum in healthy individuals (22). Previous studies using this task have revealed reduced striatal activation to reward anticipation in depressed/anhedonic individuals and increased striatal activation to both reward and punishment in anxious individuals, respectively, compared with healthy adults (17, 23, 24) making it well suited for the present study. At the onset of each trial, participants were presented with a visual cue (0.5 sec) indicating the potential outcome (reward: +$; penalty: –$; no incentive: 0$). After a variable inter-stimulus interval (2.25–3.75 sec), a red target square was briefly presented (0.15 sec). After a second variable delay (2.4–3.9 sec), visual feedback (1.25 sec) based on trial outcome (reward, penalty, or no change) was displayed and a variable intertrial interval ensued (1.5-4.5 sec). Participants were instructed to respond to the square by pressing a button as quickly as possible and that speed determines the probability of success. In order to match task difficulty across participants, the 70th percentile of each participant’s reaction time during a practice session was defined as the individual’s reaction time threshold for success. Practice session was identical to the design described above except that no feedback was displayed. In the reward condition, successful trials were associated with monetary gains ($1.98 to $2.31), whereas unsuccessful trials led to no-change. In the penalty condition, successful trials were associated with no-change, whereas unsuccessful trials were associated with monetary penalties (-$1.85 to -$2.17). No-incentive trials always ended with no-change feedback. The task included five blocks of 24 trials (8 reward, 8 penalty, and 8 no-incentive trials).

### 2.5 Imaging data acquisition

MRI data were acquired on a 3T scanner (Siemens Medical Systems, Iselin, NJ) equipped with a 32-channel head coil. fMRI data were acquired using a T2*-weighted multiband echo-planar imaging sequence (multiband factor = 3, repetition time = 1.65 sec).

### 2.6 Imaging data analyses

Both structural and functional images were processed using fMRIPrep 20.2.1 (25, 26), which is based on Nipype 1.5.1 (27, 28). Detailed information is provided in the **Supplement**.

#### 2.6.1 Structural data analyses

The T1-weighted (T1w) image was intensity bias-corrected and skull-stripped. Brain surfaces were reconstructed using recon-all (FreeSurfer 6.0.1). Intracranial volume (ICV) was calculated to correct for inter-individual differences in total brain size. All estimated volumes of *a priori* structures were exported to SPSS for statistical analyses.

#### 2.6.2 Functional data analyses

Preprocessing: The following preprocessing steps were performed – skull-strip, susceptibility distortion correction, slice time correction, spatial normalization, spatial smoothing, and automatic removal of motion artifacts using ICA-AROMa (Prium 2015). Additional motion criteria for exclusion were applied to the fMRI data (see **Supplement**).

First-level Analyses: First five volumes (8.25 sec) were discarded as dummy scans from the beginning of the task and the onset times were adjusted accordingly. First-level of single-subject fMRI data from all runs were implemented using a general linear model (GLM) in SPM. For each run, the following regressors were included in the model-reward cue, penalty cue, no-incentive (neutral) cue, successful reward feedback, unsuccessful reward feedback, successful no penalty feedback, unsuccessful penalty feedback and no-change feedback (no-incentive condition). In addition, mean time-series from the cerebrospinal fluid (CSF) and white matter (WM) masks generated from fmriprep, target, errors (i.e., when the button was pressed before the target presentation) and inter-stimulus intervals (ISIs) were included as covariates of no-interest. Each event was constructed with a hemodynamic response function, modeled using a gamma function, convolved with onset times of events and stimulus duration. A high-pass temporal filter (128 sec/0.008 Hz) was also applied to the model. Contrast maps were constructed for reward anticipation (reward vs. neutral cue) averaged across runs. The analyses were focused only on reward anticipation here due to small sample size. These contrast maps were used in region of interest (ROI)-based statistical analyses to test *a priori* hypotheses as well as for whole-brain main effects analysis evaluating brain regions affected by the task.

### 2.7 Statistical analyses

Our primary hypotheses were to investigate the effect of oligo-amenorrhea on depressive and anxiety symptoms. We also explored associated neural correlates of reward and punishment processing in the striatum (specifically caudate, putamen, and nucleus accumbens (NAc)). To this end, we focused our analyses on parsing group differences between those with eumenorrhea (EA + NA) and oligo-amenorrhea (AA). All analyses were performed in SPSS (version 22).

#### 2.7.1 Demographics and clinical questionnaires

Normality of data was determined using the Shapiro-Wilk test. Given that most data were not normally distributed, non-parametric tests (Mann-Whitney U test) were used for comparisons.

#### 2.7.2 Structural data analyses

Striatal volumes for each subject from Freesurfer outputs were exported to SPSS for analyses. To test striatal volumes, a Group (oligo-amenorrheic/eumenorrheic) x ROI (Caudate, Putamen, NAc) x Hemisphere (Left, Right) repeated measures ANOVA was run controlling for age and Intracranial volume (ICV). Significant interactions were followed by individual *post-hoc* tests.

#### 2.7.3 Functional data analyses

To investigate the neural correlates of reward and punishment anticipation and to validate the task, a one-sample t-test was conducted across all subjects. Cluster correction at p < 0.05 family-wise error (FWE) with an initial voxel forming threshold of p < 0.001 was utilized. ROI Analyses: To test *a priori* hypotheses that oligo-amenorrhea would affect reward-related activation in the striatum, we created left and right anatomical NAc, caudate, and putamen ROIs from the FSL Harvard-Oxford Subcortical Atlas using a 40% probability threshold. For each subject, parameter estimates from each of the ROIs were extracted from reward and penalty anticipation contrast maps and were entered into SPSS (version 22). Throughout the analyses, data were inspected for the presence of outliers. Values that exceeded three times the inter-quartile range (the difference between the third and first quartile) of mean parameter estimates were deemed to be outliers and were further investigated to identify if they were due to motion, registration error, or other sources of artifacts. If no problems could be identified and corrected, outlier data points were removed. After inspection for outliers, a repeated measures ANOVA with Group (amenorrheic/eumenorrheic as a between-subject factor and Hemisphere (Right/Left) and Valence (Cue Reward – Cue Neutral, CR_CN/Cue Punishment – Cue Neutral, CP_CN) as within-subject factors was run for each ROI to investigate effects of amenorrhea on reward and punishment anticipation.

## 3 Results

### 3.1 Demographics and clinical questionnaires

**Table 1** summarizes participant characteristics in the two groups. AA had lower BMI and were significantly older at menarche compared with the eumenorrhea group (t=-2.85, p=0.005; t=2.65, p=0.006, respectively). As expected, AA had higher anxiety (measured by STAI Trait scores, t=2.01, p=0.03) and depressive symptomatology (higher anhedonic depression measured by MASQ scores, t=2.04, p=0.04) and lower estradiol levels (t=-2.02, p=0.004) compared with those with eumenorrhea (see **Table 2**).

**Table 1:** Participant characteristics by menstrual groups.

	Oligo-amenorrheic group (n=24) Median (IQR)	Eumenorrheic group (n=27)	P value	Effect size Cohen’s D
**Age (years)**	20.6 (19.0-22.6)	20.6 (19.2-23.7)	0.578	0.14
**BMI (kg/m^2^)**	20.3 (18.8-21.5)	21.9 (19.6-23.5)	**0.005**	0.8
**Percent median BMI**	93.9 (90.0-100.0)	101.8 (91.7-108.6)	**0.008**	0.27
Activity (hours/week) *	8.5 (6.7-11.3)	4.3 (0.4-9.9)	**0.011**	0.56
Age of menarche (years) *	13.0 (12.5-15.0)	12 (12-13)	**0.006**	0.74

Median and IQR (25^th^-75^th^) Mann-Whitney U Test was used for the overall p value; P values <0.05 are bolded. *Data only available for this variable in 23 oligo-amenorrheic participants.

**Table 2:** Questionnaires and hormones by menstrual groups (to be removed, added to manuscript).

	Oligo-amenorrheic group (n=23)	Eumenorrheic group (n=26)	P value	Effect Size Cohen’s d
**BDI-II** ^a^	4 (0-13.8)	1.5 (0-3)	0.19	0.453
**STAI** ^a^	32.5 (27.0-51.0)	27 (25-36.3)	**0.03**	0.583
**MASQ (Anhedonic Depression)**	51 (42.0-64.0)	42.0 (35.3-48.3)	**0.04**	0.583
**Estradiol (pg/ml)** ^b^	24.4 (16.5-38)	52.5 (32.5-107.0)	**0.004**	0.835

Median and IQR (25th-75th). Mann-Whitney U Test was used for the overall p value. BDI-II, Beck Depression Inventory-II; STAI, State-Trait Anxiety; MASQ, Mood and Anxiety Symptom Questionnaire; P values <0.05 are bolded.

a Data only available for these variables in n = 22 (oligo-amenorrheic group).

b Data only available in 9 oligo-amenorrheic and 23 eumenorrheic participants.

### 3.2 Structural analyses

**Figure 1:**
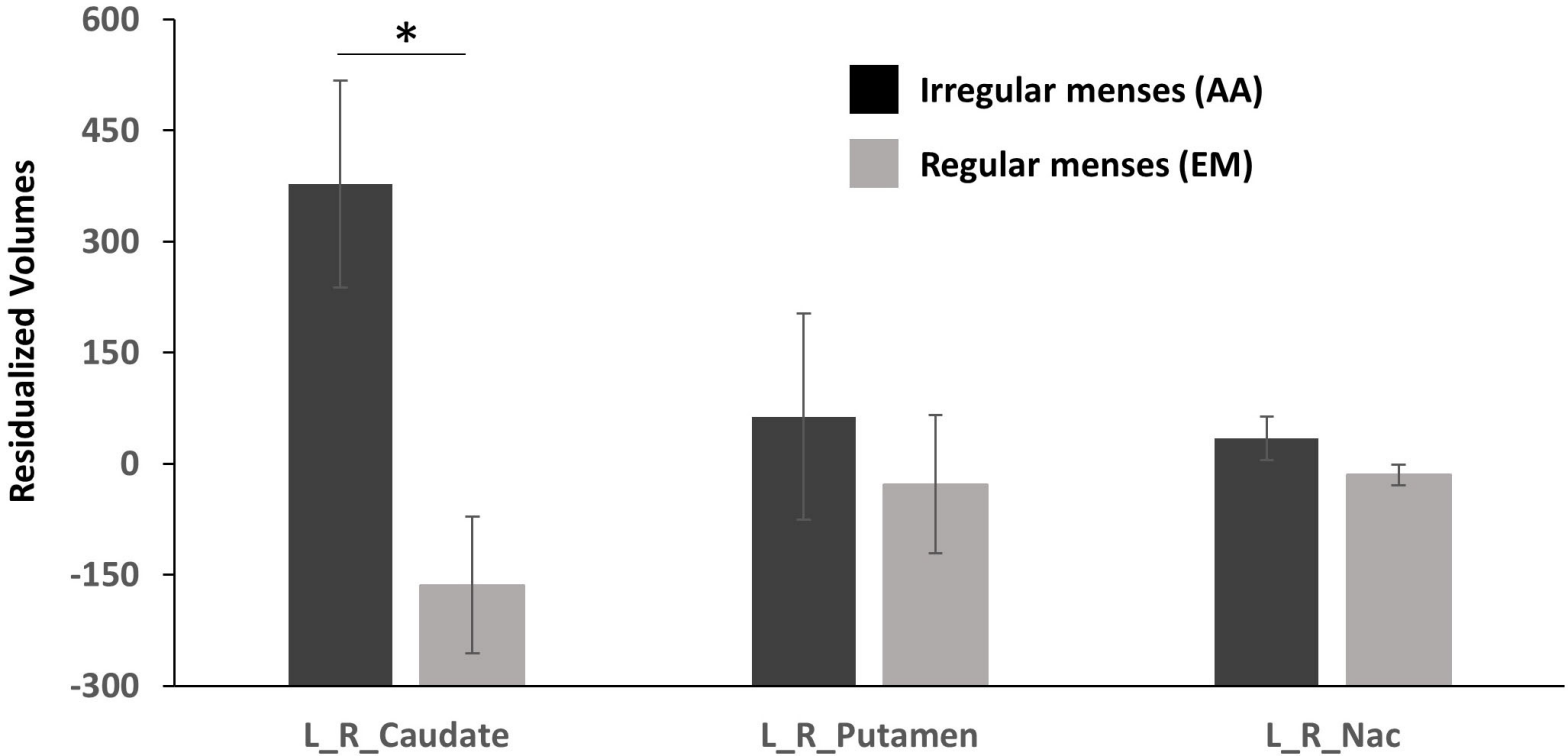
Residualized volumetric (corrected for age and ICV) differences in the caudate, putamen, and nucleus accumbens (NAc) between participants with oligo-amenorrhea and eumenorrhea. Error bars indicate standard error. *p < 0.05.

Striatal ROI: Group (oligo-amenorrhea, eumenorrhea) x ROI (Caudate, Putamen, NAc) x Hemisphere (Left, Right) repeated measures ANOVA controlling for ICV and age revealed a significant ROI x Group [F(1,2)=4.6, p=0.014, η2=0.14] and a main effect of Group [F(1,29)=5.49, p=0.026, η2=0.16], but no effects of ROI or Hemisphere. Further *post-hoc* analyses adjusted for Bonferroni correction revealed that AA had higher caudate volumes compared to the eumenorrheic group [F(1, 29)=9.930, p=0.004, η^2^=0.26]. However, no significant differences were observed for putamen [F(1, 29)=0.28, p>0.5, η^2^=0.009] or NAc volumes (F(1, 29)=2.81, p=0.11, η^2^=0.09; see **Figure 1**].

### 3.3 Functional monetary incentive delay analyses

Two participants (two EA) were excluded as they had >10% of trials classified as motion outliers based on motion criteria mentioned in the Data Quality section in the **Supplement**. Whole brain analysis of reward and punishment anticipation across all subjects was conducted to validate the task. Consistent with other studies, significant activations were observed in the bilateral striatum, mid-cingulate and orbitofrontal cortex (see **Supplement Figures S1, S2** and **Tables S1, S2**).

#### 3.3.1 Caudate

**Figure 2:**
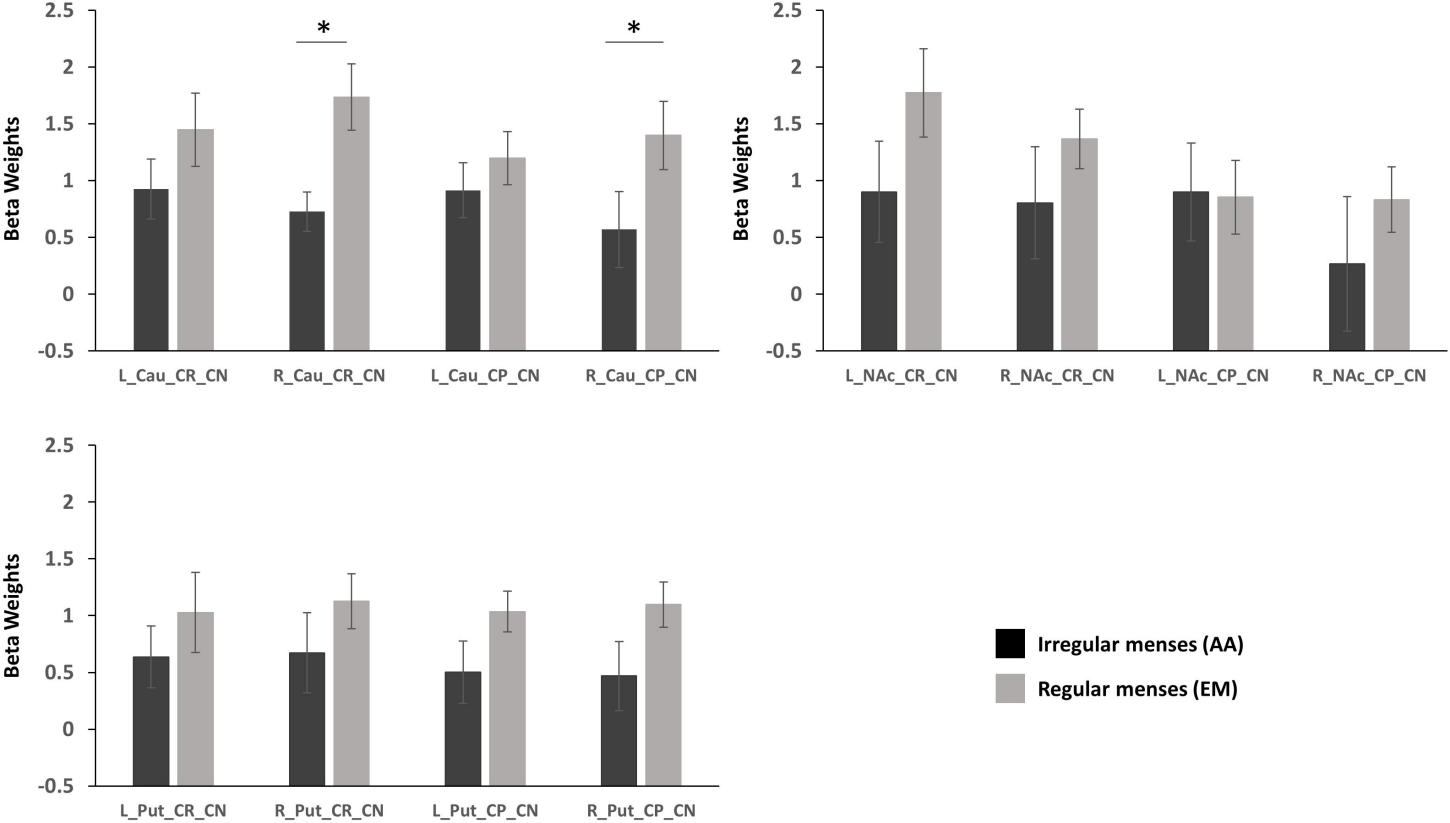
Standardized parameter estimates extracted from functional ROIs during reward anticipation (Cue Reward – Cue Neutral, CR_CN) and punishment activation (Cue Punishment – Cue Neutral, CP_CN) in the caudate (Cau), putamen (Put), and nucleus accumbens (NAc) in participants with oligo-amenorrhea and eumenorrhea. Error bars indicate standard error. *p < 0.05.

Group (oligo-amenorrhea, eumenorrhea) x Hemisphere (Left, Right) x Valence (CR_CN/CP_CN) repeated measures ANOVA in the caudate revealed a significant Group x Hemisphere effect [F(1,29)=5.61, p=0.025, η^2^=0.16). *Post-hoc* analyses adjusted for Bonferroni correction revealed that this effect was mainly driven by differences in the right caudate, with lower activation observed in the AA group (p=0.036, η^2^=0.14) during both reward and punishment anticipation. No significant differences were observed in the left caudate (p=0.29, η^2^=0.04; **Figure 2**).

#### 3.3.2 Nucleus accumbens

A Group x Valence x Hemisphere interaction was observed [F(1,29)=4.7, p=0.038, η^2^=0.14], but further *post-hoc* analyses revealed no significant effects.

#### 3.3.3 Putamen

No main effects or interactions were observed in the putamen during reward or punishment anticipation.

### 3.4 Sensitivity analyses

To ensure that structural and functional differences between oligo-amenorrheic and eumenorrheic individuals were not driven by depression/anxiety, physical activity, we repeated the analyses controlling for these variables.

Controlling for depression and anxiety scores: Anhedonic depression and anxiety scores data were not available for 1 oligo-amenorrheic and 1 eumenorrheic individual. The higher caudate volume and lower caudate activation to reward/punishment anticipation observed in AA compared to the EM group remained significant even after controlling for anhedonic depression/anxiety scores [Caudate volume - F(1, 25)=7.234, p=0.013, η^2^=0.22; Caudate functional activation – F(1, 25)=4.778, p=0.038, η^2^=0.16].

Controlling for physical activity (Average hours/week for the past year): Physical activity data were not available for 1 oligo-amenorrheic individual. AA had higher caudate volumes compared to the EM group after controlling for physical activity [F(1, 27)=6.060, p=0.021, η^2^=0.18]. However, after controlling for physical activity, the decreased activation in the right caudate during reward and punishment anticipation in AA reached significance only at a trend level [F(1,270 = 3.76, p = 0.063, η^2^=0.12], suggesting physical activity may play a small role in this effect.

Sensitivity analyses for age at menarche (as age at menarche was different between groups) and BMI (as a proxy for nutritional status) are provided in the **Supplement**.

## 4 Discussion

Our study adds to the sparse literature on mood and anxiety and related structural and functional reward-related changes in oligo-amenorrheic athletes. Consistent with our hypothesis that estrogen deficiency is associated with mood and anxiety alterations; depressive and anxiety symptoms were significantly higher in female athletes with oligo-amenorrhea compared to young women with eumenorrhea. Further, bilateral caudate volume was higher in AA compared with the eumenorrheic group even after adjusting for age and intracranial volume. We also found that AA had decreased activation in the right caudate during anticipation of reward and punishment in the monetary incentive delay task, suggesting that estrogen status may play a role in modulating mood and anxiety. These findings persisted even after we controlled for possible confounders.

Effects of gonadal hormones on mood have been demonstrated in ovariectomized mice, who show symptoms of increased anxiety and reluctance to venture into the center of an open area to seek out food (29) as well as depressive behavior (30). Interestingly, such behaviors normalize after estrogen replacement (30, 31). Women with estrogen deficiency (e.g., post-menopause, Turner syndrome, primary ovarian insufficiency) have a higher prevalence of mood alterations and anxiety compared with the general population (4, 5, 32). Further, functional imaging studies in normally menstruating adult women have described a correlation between estradiol levels and blood oxygen level dependent (BOLD) signal activation in the amygdala-hippocampal region following a reward, indicating that gonadal steroids may play a role in modulation of reward processing (33). Importantly, in a prior study, we showed that hypogonadal adolescent girls with anorexia nervosa (AN) and FHA have an improvement in trait anxiety following estrogen replacement (12).

Consistent with these reports, in the current study, AA exhibited higher scores for anhedonic depression and anxiety compared to the eumenorrheic group. Athletic amenorrhea is a form of FHA, which is characterized by increased depressive symptoms and difficulty dealing with stressors. These individuals tend to display dysfunctional attitudes such as perfectionistic behavior, extra attention to the judgments of others and unrealistic expectations compared to eumenorrheic individuals (10, 11). In one study of 21 healthy controls, 18 amenorrheic women with AN, and 13 normal-weight women with FHA, women with AN and FHA had higher scores for anxiety and depressive symptomology based on the Hamilton Rating Scale for Anxiety (HAM-A) and Depression (HAM-D) than the healthy controls (34). It is important to note that, although depressive and anxiety symptoms were higher in the group with oligo-amenorrhea, mean scores in our study did not reach the level of clinical significance for a diagnosis of anxiety or depression. Exercise, by virtue of being a mood elevator, may have offered some protection to these individuals.

The finding that AA demonstrated increased volume of the caudate region compared to eumenorrheic participants suggests that estrogen may play an important role in volume reductions in specific brain areas during puberty. A longitudinal pediatric neuroimaging study by Giedd et al. indicated that gray matter in the frontal lobe increased linearly during pre-adolescence with maximum size noted before puberty, followed by a decline post-adolescence resulting in a net decrease in volume. Gray matter volume in the parietal lobes followed a similar pattern for changes in volume, except that the slopes were steeper pre- and post-adolescence. This structural change in both lobes peaked one year earlier in females than males, suggesting that maturational volume reductions in these regions may be potentially driven by the influence of estrogen (35). Of note, Lepage et al. evaluated developmental brain morphology in adolescents with Turner syndrome (TS), a condition characterized by a lack of endogenous estrogen. This study compared 30 girls with TS with 21 age-matched healthy female controls, and showed that hypoestrogenic girls with TS have significantly higher caudate volume (similar to findings in athletes with oligo-amenorrhea in our study) suggesting that this may result from delayed maturational involution linked to the lack of estrogen during adolescence (36).

Rodent studies indicate that estrogen has dopamine agonist actions and promotes dopamine secretion in the striatum and thereby improves reward sensitivity (37, 38). Functional neuroimaging studies in humans have yielded similar findings. During the mid-follicular phase of the menstrual cycle, estradiol levels positively correlate with brain activity in the amygdala and hippocampal complex during reward anticipation (33). In a more recent study, Macoveneau et al. examined reward processing in 58 women randomized to GnRH analogues (which block sex steroid production) and placebo (39). Compared to participants on placebo, the GnRH analogue group showed reduced activation in the amygdala to monetary gains suggesting that sex steroids may modulate reward processing (39). Of note, these participants also had increased depressive symptoms. Similarly, estrogen replacement in perimenopausal women resulted in increased response in the striatum during reward anticipation relative to placebo (40). Previous studies have also demonstrated hypoactivation to gains as well as penalties in patients with depression compared with healthy individuals (24). Our finding of decreased activation in the caudate regions for reward and punishment anticipation (functional MRI task) is in line with these findings and with our hypothesis that estrogen deficiency may blunt the response to reward processing.

The main limitation of our study is the small number of participants with MRI scans. This resulted in the eumenorrheic athlete and non-athlete groups being combined, as they both have regular menses (and also because these two groups were similar for STAI and MASQ scores). The significance of reduced caudate activation in AA vs EM (p=0.025) did not survive multiple corrections applied for 3 ROIs (p = 0.05/3 = 0.017), possible driven by small sample size. However, it is important to note that our results are characterized by moderate effect sizes. A few participants could not be included due to technical difficulties with MRI processing and image acquisition, and it will be important to replicate our findings in a larger sample. The second limitation relates to the relatively normal scores of anhedonic depression and anxiety in study participants, indicating no increase in a clinical diagnosis of depression or anxiety, notwithstanding higher mean STAI and MASQ anhedonic depression scores in oligo-amenorrheic vs. eumenorrheic women. Thus, higher mean scores on these questionnaires do not necessarily translate to a clinical diagnosis of anxiety or depression (known to be associated with structural brain changes). This was further corroborated by our sensitivity analyses that showed the significances for caudate functional and structural alterations persisted even after controlling for anhedonic depression and anxiety scores. Similarly, controlling for physical activity and menarchal age did not change our results for structural differences between groups. However, the reduced caudate activation in the AA group was significant only at a trend level after controlling for physical activity, suggesting that exercise, by virtue of being a mood elevator, may have offered some (albeit a small) protection to these individuals. The lack of significance may have been due to the small sample size in our exploratory analyses. Lack of data regarding caloric intake and the use estrogen status based on menstrual status (as estradiol levels were not available in all individuals and do not necessarily reflect the chronicity of estrogen exposure as they are cycle phase dependent) are other limitations that need to be addressed in future studies. Although our data provide important preliminary evidence that estrogen status may play a role in brain structure and function, we cannot determine causality as these are cross sectional studies. These findings underscore the need to identify young athletes with estrogen deficiency and to evaluate the implications of these findings on psychiatric endpoints. From a clinical perspective, identifying comorbidities related to estrogen status may help propose early interventions involving improved diet, reduced activity and psychological counseling and may help improve outcomes.

## 5 Conclusion

Oligo-amenorrheic athletes have greater anxiety and depressive symptoms compared with young women with eumenorrhea. Oligo-amenorrheic athletes also have increased caudate volumes, which may be related to delayed maturational decrease in cortical volumes seen in estrogen deficiency states. In addition, relative to eumenorrheic athletes and non-athletes, oligo-amenorrheic athletes demonstrate blunted responses to rewards. These findings require validation in larger studies.

## Data availability statement

The original contributions presented in the study are included in the article/**Supplementary Material**. Further inquiries can be directed to the corresponding author.

## Ethics statement

The studies involving human participants were reviewed and approved by Institutional Review Board MGH. Written informed consent to participate in this study was provided by the participants’ legal guardian/next of kin.

## Author contributions

PK and SN analyzed the data, CB, PK and SN interpreted the data. CB, PK, SN, and MM were contributors in writing the manuscript. FP, KA, KE, DP, and MM edited and reviewed and edited the manuscript. CB and MM conceptualized and obtained the funding for the study. All authors read and approved the final manuscript.
