# Supplementary material for: Depressive and anxiety symptoms, and neural correlates of reward and punishment anticipation in female athletes with amenorrhea
Source: Front Endocrinol (Lausanne). 2023 May 18;14:976050. doi: 10.3389/fendo.2023.976050 (PMC10233051; doi:10.3389/fendo.2023.976050)
Supplement: Supplementary file 1 [file DataSheet_1.docx]

Supplementary Material

**Imaging Data Acquisition:** The MRI data were acquired on a Siemens 3T scanner (Siemens Medical Systems, Iselin, NJ, USA) equipped with a 32-channel head coil. High-resolution structural data were acquired using a T1-weighted magnetization-prepared rapid acquisition with gradient multi echo (MPRAGE) imaging sequence with the following acquisition parameters: repetition time (TR) = 2200 ms; echo times (TE) = 1.54, 3.36, 5.18 and 7 ms; field of view = 230 mm; voxel dimensions = 1.2 x 1.2 x 1.2 mm; 144 slices. Functional MRI data were acquired using a gradient echo T2*- weighted multiband echo planar imaging sequence (Feinberg et al., 2010; Moeller et al., 2010; Xu et al., 2013) with the following acquisition parameters: repetition time (TR) = 1650 ms; echo time (TE) = 30 ms; field of view = 110 mm; voxel dimension = 2.0 x 2.0 x 2.4 mm; flip angle = 75degree, 75 interleaved slices and a multiband factor of 3.

**Imaging Data Analyses:** Both structural and functional images were processed using fMRIPrep 20.2.1 (Esteban, Markiewicz, et al., 2018; Esteban, Blair, et al., 2018; RRID:SCR_016216), which is based on Nipype 1.5.1 (Gorgolewski et al., 2011, 2018).

**Structural Data Analyses:** The T1-weighted (T1w) image was corrected for intensity non-uniformity (INU) with N4BiasFieldCorrection (Tustison et al. 2010), distributed with ANTs 2.3.3 (Avants et al. 2008, RRID:SCR_004757), and used as T1w-reference throughout the workflow. The T1w-reference was then skull-stripped with a Nipype implementation of the antsBrainExtraction.sh workflow (from ANTs), using OASIS30ANTs as target template. Brain tissue segmentation of cerebrospinal fluid (CSF), white matter (WM) and gray matter (GM) was performed on the brain-extracted T1w using fast (FSL 5.0.9, RRID:SCR_002823, Zhang, Brady, and Smith 2001). Brain surfaces were reconstructed using recon-all (FreeSurfer 6.0.1, RRID:SCR_001847, Dale, Fischl, and Sereno 1999). Intracranial volume (ICV) was also calculated to correct for inter-individual differences in total brain size. All estimated volumes of *a priori* structures were exported to SPSS for statistical analyses.

**Functional Data Analyses:** Preprocessing: The following preprocessing was performed. First, a reference volume and its skull-stripped version were generated using a custom methodology of fMRIPrep. A deformation field to correct for susceptibility distortions was estimated based on fMRIPrep’s fieldmap-less approach. The deformation field is that resulting from co-registering the BOLD reference to the same-subject T1w-reference with its intensity inverted (Wang et al. 2017; Huntenburg 2014). Registration was performed with antsRegistration (ANTs 2.3.3), and the process regularized by constraining deformation to be nonzero only along the phase-encoding direction, and modulated with an average fieldmap template (Treiber et al. 2016). Based on the estimated susceptibility distortion, a corrected EPI (echo-planar imaging) reference was calculated for a more accurate co-registration with the anatomical reference. The BOLD reference was then co-registered to the T1w reference using bbregister (FreeSurfer) which implements boundary-based registration (Greve and Fischl 2009). Co-registration was configured with six degrees of freedom. Head-motion parameters with respect to the BOLD reference (transformation matrices, and six corresponding rotation and translation parameters) were estimated before any spatiotemporal filtering using mcflirt (FSL 5.0.9, Jenkinson et al. 2002). BOLD runs were slice-time corrected using 3dTshift from AFNI 20160207 (Cox and Hyde 1997, RRID:SCR_005927). The BOLD time-series were resampled onto the following surfaces (FreeSurfer reconstruction nomenclature): fsaverage. The BOLD time-series (including slice-timing correction when applied) were resampled onto their original, native space by applying a single, composite transform to correct for head-motion and susceptibility distortions. These resampled BOLD time-series will be referred to as preprocessed BOLD in original space, or just preprocessed BOLD. The BOLD time-series were resampled into standard space, generating a preprocessed BOLD run in MNI152NLin6Asym space. First, a reference volume and its skull-stripped version were generated using a custom methodology of fMRIPrep. Automatic removal of motion artifacts using independent component analysis (ICA-AROMA, Pruim et al. 2015) was performed on the preprocessed BOLD on MNI space time-series after removal of non-steady state volumes and spatial smoothing with an isotropic, Gaussian kernel of 6mm FWHM (full-width half-maximum). Corresponding “non-aggressively” denoised runs were produced after such smoothing. All resamplings can be performed with a single interpolation step by composing all the pertinent transformations (i.e. head-motion transform matrices, susceptibility distortion correction when available, and co-registrations to anatomical and output spaces). Gridded (volumetric) resamplings were performed using antsApplyTransforms (ANTs), configured with Lanczos interpolation to minimize the smoothing effects of other kernels (Lanczos 1964). Non-gridded (surface) resamplings were performed using mri_vol2surf (FreeSurfer). Many internal operations of fMRIPrep use Nilearn 0.6.2 (Abraham et al. 2014, RRID:SCR_001362), mostly within the functional processing workflow. For more details of the pipeline, see the section corresponding to workflows in fMRIPrep’s documentation.

**Data Quality assurance:** Several confounding time-series were calculated based on the preprocessed BOLD: framewise displacement (FD), DVARS and three region-wise global signals. FD and DVARS were calculated for each functional run, using their implementations in Nipype (following the definitions by Power et al. 2014). The three global signals were extracted within CSF, WM, and whole-brain masks. Frames that exceeded a threshold of 0.5 mm FD or 1.5 standardized DVARS were annotated as motion outliers. Subjects that had >10% of trials classified as motion outliers were excluded from further analyses.

**RESULTS:**

**Whole-brain analyses:** Consistent with other studies, significant activations were observed in the bilateral striatum, mid cingulate, orbitofrontal cortex during both reward and punishment activation (See Supplemental Figures 1 & 2 and Tables S1 & S2).


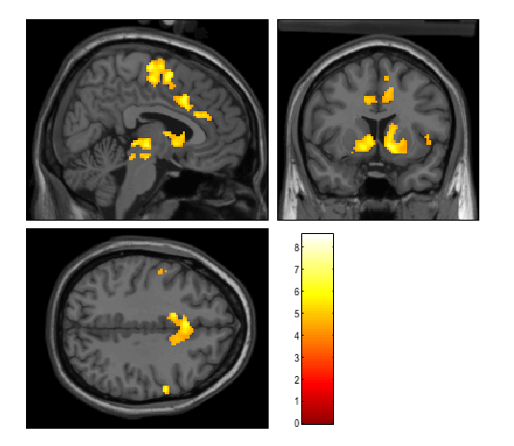


**Supplemental Figure 1:** Whole-brain main effects for whole group during reward anticipation (n = 31). Clusters significant at FWE p < 0.05 with initial cluster forming threshold p < 0.0001. Significant clusters reported in Supplementary Table 1A.


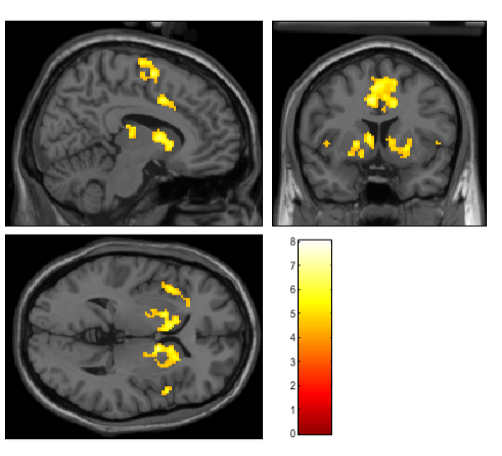


**Supplemental Figure 2:** Whole-brain main effects for whole group during punishment anticipation (n = 31). Clusters significant at FWE p < 0.05 with initial cluster forming threshold p < 0.0001. Significant clusters reported in Supplementary Table 1B.

**Supplementary Table:** MNI peak Coordinates of brain regions during (A) reward anticipation (Cue Reward – Cue Neutral) and (B) punishment activation (Cue Punishment – Cue Neutral). p < 0.05 Family Wise Error (FWE) cluster corrected, with an initial cluster forming threshold of p = 0.0001.

**Table S1. Cue Reward – Cue Neutral**

| **Brain Region** | **Cluster size** | **MNI (x, y, z)**  **(peak)** | **Z score**  **(peak)** | **Cluster p (FWE)** |
| --- | --- | --- | --- | --- |
| Supplementary Motor Cortex  Brain Stem  Right Striatum  Left Striatum  Frontal Operculum Cortex  Precentral Gyrus  Orbitofrontal Cortex | 6391  4224  140  690  97  78  93 | 0, -6, 70  6, -30, -8  10, 16, 2  -14, 12, -4  42, 14, 6  54, 0, 40  -30, 28, -2 | 6.05  5.51  4.99  4.88  4.81  4.75  4.42 | <0.001  <0.001  0.002  <0.001  0.016  0.045  0.020 |

**Table S2. Cue Punishment – Cue Neutral**

| **Brain Region** | **Cluster size** | **MNI (x, y, z)**  **(peak)** | **Z score**  **(peak)** | **Cluster p (FWE)** |
| --- | --- | --- | --- | --- |
| Thalamus  Right Striatum  Left Striatum  Supplementary Motor Cortex  Cerebellum  Right Frontal Operculum  Left Frontal Operculum Cortex  Cerebellum  Precentral Gyrus  Supramarginal Gyrus | 116  579  504  2758  254  134  246  57  169  86 | -14, -22, 16  12, 2, -2  -14, 12, -4  -6, -8, 60  20, -50, -24  42, 16, 6  -32, 24, 10  0, -58, -36  32, -12, 54  -60, -44, 32 | 5.82  5.82  5.70  5.55  5.31  5.16  5.15  5.05  4.77  4.36 | <0.001  <0.001  <0.001  <0.001  <0.001  <0.001  <0.001  0.001  <0.001  <0.0001 |

**Sensitivity analyses controlling for age at menarche and BMI**

Controlling for age at menarche (as age at menarche was different between groups): AA had higher caudate volumes compared to the EM group after controlling for age of menarche [F(1, 29)=13.01, p=0.001, η^2^=0.31]. Similarly, the decreased activation in the right caudate during reward and punishment activation in AA remained significant [F(1,26)=5.3, p = 0.012, η^2^ = 0.29], suggesting age of menarche did not affect these findings.

Controlling for BMI (as a proxy for nutritional status): AA had higher caudate volumes compared to the EM group after controlling for BMI [F(1, 29)=9.9, p=0.004, η2=0.25]. Similarly, the decreased activation in the right caudate during reward and punishment activation in AA trended significant [F(1,26)=5.9,p = 0.053, η2=0.20], suggesting that nutritional status can play a small role on these findings.

**Correlations with Estradiol**

We did not find a significant correlation between estradiol levels and caudate volume (r = 0.023, p =0.905, n = 32) or caudate activation during reward and punishment anticipation (r = 0.25, p = 0.19, n = 30) in the subset of participants who had estradiol levels.

**Subgroup Analyses:**

**Table S3: Sub analyses based on athlete group- Participant characteristics:**

|  | **AA**  **(n=24)** | **EA**  **(n=14)** | **NA**  **(n=13)** | **P value** | **AA vs.**  **NA** | **EA vs. NA** | **AA vs. EA** |
| --- | --- | --- | --- | --- | --- | --- | --- |
| **Age (years)** | 20.6 (19.0-22.6) | 20.6 (19.1-23.1) | 19.9 (19.1-24.4) | 0.79 | na | na | na |
| **BMI (kg/m^2^)** | 20.3 (18.8-21.5) | 22.7 (20.6-23.5) | 21.8 (19.5-23.0) | **0.01** | 0.09 | 0.29 | **0.003** |
| **Percent Ideal BMI** | 93.9 (90.0-100.0) | 104.3 (95.8-108.9) | 100.6 (89.6-105.8) | **0.02** | 0.16 | 0.22 | **0.004** |
| **Activity (hours/week)*** | 8.5 (6.7-11.3) | 9.8 (6.7-13.5) | 0.35 (0-1.1) | **<0.001** | **<0.001** | **<0.001** | 0.67 |
| **Age of menarche (years) *** | 13.0 (12.5-15.0) | 12 (12-13.3) | 12 (11.5-12.75) | **0.019** | **0.01** | 0.57 | 0.047 |

Median and Interquartile range shown. Kruskal-Wallis test was used for the overall p value. Steel-Dwass test was used for the 3-group comparison. P values < 0.05 are bolded. For insignificant p values post-hoc comparisons were not done; na, AA: Oligo-amenorrheic athletes, EA: Eumenorrheic athletes, and NA: Non-athletes. *****Data only available for this variable in 23 AA participants.

**Table S4: Sub analyses based on athlete group- Questionnaires and estradiol:**

|  | AA  (n=23) | EA  (n=14) | NA  (n=13) | P value | AA vs.  NA | EA vs. NA | AA vs. EA |
| --- | --- | --- | --- | --- | --- | --- | --- |
| BDI-II ^a^ | 4 (0-13.8) | 2 (1-3) | 1 (0-2) | 0.15 | na | na | na |
| STAI ^a^ | 32.5 (27.0-51.0) | 27 (25-35.5) | 27 (25-37.5) | 0.11 | na | na | na |
| MASQ (Anhedonic Depression) ^b^ | 51 (42.0-64.0) | 40 (34.5-55.5) | 43 (34-47.5) | 0.11 | na | na | na |
| Estradiol (pg/ml)^c^ | 24.4 (16.5-38) | 50.8 (32.7-87.7) | 52.5 (24.9-136) | **0.02** | 0.035 | 0.68 | **0.006** |

Median and Interquartile range (IQR) shown Kruskal-Wallis test was used for the overall p value. Steel-Dwass test was used for the 3-group comparison. P values < 0.05 are bolded. For insignificant p values post hoc comparisons were not done; na, AA: Oligo-amenorrheic athletes, EA: Eumenorrheic athletes and NA: Non-athletes. ^a^Data only available for these variables in n = 22 in AA group and 13 in EA group. ^b^Data were available only in 13 EA. ^c^Data only available in 9 participants in AA, and 9 participants in NA.

**Table S5: Sub analyses based on athlete group - Structural and functional data:**

|  | AA | EA | NA | P value | AA vs. HC | EA vs. HC | AA vs. EA |
| --- | --- | --- | --- | --- | --- | --- | --- |
| Caudate | 377.56±139.89  N=10 | -118.45±141.43  N=13 | -223.58±116.63  N=10 | **0.014** | 0.020 | n.s | 0.047 |
| Right Caudate CR-CN/CP_CN (avg) | 0.65±0.23  N=10 | 1.97±0.35  N=11 | 1.12±0.37  N=10 | **0.023** | n.s | n.s | 0.022 |

Mean±S.E.M. Univariate ANOVA with 3 groups controlled for age and ICV. P values < 0.05 are bolded. AA: Oligo-amenorrheic athletes, EA: Eumenorrheic athletes, and NA: Non-athletes. n.s: non-significant

**Structural Analyses:** There was an overall main effect of group in caudate volume with three subgroups (EA, AA, and NA) and controlled for age and ICV, [F(1,28)=5.0, p = 0.014, η^2^ = 0.26]. On further post-hoc analyses (Bonferroni correction for multiple comparisons applied), there was a significant group difference between AA vs. NA (p =0.020) and AA vs. EA (p=0.047), but no difference between NA vs. EA (n.s.) with AA having higher caudate volume compared with EA and NA.

**Functional Analyses:** A valence (Reward, Punishment) x Group ANOVA of right caudate activation revealed a main effect of group, [F(1,28) = 4.35, p = 0.023, η^2^ = 0.24]. On further post-hoc analyses (Bonferroni correction for multiple comparisons applied), AA group was significantly lower than EA (p = 0.022), with NA numerically in between AA and EA.

**References**

Abraham, Alexandre, Fabian Pedregosa, Michael Eickenberg, Philippe Gervais, Andreas Mueller, Jean Kossaifi, Alexandre Gramfort, Bertrand Thirion, and Gael Varoquaux. 2014. “Machine Learning for Neuroimaging with Scikit-Learn.” Frontiers in Neuroinformatics 8. https://doi.org/10.3389/fninf.2014.00014.

Avants, B.B., C.L. Epstein, M. Grossman, and J.C. Gee. 2008. “Symmetric Diffeomorphic Image Registration with Cross-Correlation: Evaluating Automated Labeling of Elderly and Neurodegenerative Brain.” Medical Image Analysis 12 (1): 26–41. https://doi.org/10.1016/j.media.2007.06.004.

Behzadi, Yashar, Khaled Restom, Joy Liau, and Thomas T. Liu. 2007. “A Component Based Noise Correction Method (CompCor) for BOLD and Perfusion Based fMRI.” NeuroImage 37 (1): 90–101. https://doi.org/10.1016/j.neuroimage.2007.04.042.

Cox, Robert W., and James S. Hyde. 1997. “Software Tools for Analysis and Visualization of fMRI Data.” NMR in Biomedicine 10 (4-5): 171–78. https://doi.org/10.1002/(SICI)1099-1492(199706/08)10:4/5<171::AID-NBM453>3.0.CO;2-L.

Dale, Anders M., Bruce Fischl, and Martin I. Sereno. 1999. “Cortical Surface-Based Analysis: I. Segmentation and Surface Reconstruction.” NeuroImage 9 (2): 179–94. https://doi.org/10.1006/nimg.1998.0395.

Esteban, Oscar, Ross Blair, Christopher J. Markiewicz, Shoshana L. Berleant, Craig Moodie, Feilong Ma, Ayse Ilkay Isik, et al. 2018. “FMRIPrep.” Software. Zenodo. https://doi.org/10.5281/zenodo.852659.

Esteban, Oscar, Christopher Markiewicz, Ross W Blair, Craig Moodie, Ayse Ilkay Isik, Asier Erramuzpe Aliaga, James Kent, et al. 2018. “fMRIPrep: A Robust Preprocessing Pipeline for Functional MRI.” Nature Methods. https://doi.org/10.1038/s41592-018-0235-4.

Evans, AC, AL Janke, DL Collins, and S Baillet. 2012. “Brain Templates and Atlases.” NeuroImage 62 (2): 911–22. https://doi.org/10.1016/j.neuroimage.2012.01.024.

Fonov, VS, AC Evans, RC McKinstry, CR Almli, and DL Collins. 2009. “Unbiased Nonlinear Average Age-Appropriate Brain Templates from Birth to Adulthood.” NeuroImage 47, Supplement 1: S102. https://doi.org/10.1016/S1053-8119(09)70884-5.

Gorgolewski, K., C. D. Burns, C. Madison, D. Clark, Y. O. Halchenko, M. L. Waskom, and S. Ghosh. 2011. “Nipype: A Flexible, Lightweight and Extensible Neuroimaging Data Processing Framework in Python.” Frontiers in Neuroinformatics 5: 13. https://doi.org/10.3389/fninf.2011.00013.

Gorgolewski, Krzysztof J., Oscar Esteban, Christopher J. Markiewicz, Erik Ziegler, David Gage Ellis, Michael Philipp Notter, Dorota Jarecka, et al. 2018. “Nipype.” Software. Zenodo. https://doi.org/10.5281/zenodo.596855.

Greve, Douglas N, and Bruce Fischl. 2009. “Accurate and Robust Brain Image Alignment Using Boundary-Based Registration.” NeuroImage 48 (1): 63–72. https://doi.org/10.1016/j.neuroimage.2009.06.060.

Huntenburg, Julia M. 2014. “Evaluating Nonlinear Coregistration of BOLD EPI and T1w Images.” Master’s Thesis, Berlin: Freie Universität. http://hdl.handle.net/11858/00-001M-0000-002B-1CB5-A.

Jenkinson, Mark, Peter Bannister, Michael Brady, and Stephen Smith. 2002. “Improved Optimization for the Robust and Accurate Linear Registration and Motion Correction of Brain Images.” NeuroImage 17 (2): 825–41. https://doi.org/10.1006/nimg.2002.1132.

Klein, Arno, Satrajit S. Ghosh, Forrest S. Bao, Joachim Giard, Yrjö Häme, Eliezer Stavsky, Noah Lee, et al. 2017. “Mindboggling Morphometry of Human Brains.” PLOS Computational Biology 13 (2): e1005350. https://doi.org/10.1371/journal.pcbi.1005350.

Lanczos, C. 1964. “Evaluation of Noisy Data.” Journal of the Society for Industrial and Applied Mathematics Series B Numerical Analysis 1 (1): 76–85. https://doi.org/10.1137/0701007.

Moeller, S., Yacoub, E., Olman, C. A., Auerbach, E., Strupp, J., Harel, N., & Uğurbil, K. (2010). Multiband multislice GE-EPI at 7 tesla, with 16-fold acceleration using partial parallel imaging with application to high spatial and temporal whole-brain fMRI. Magn Reson Med, 63(5), 1144–53

Power, Jonathan D., Anish Mitra, Timothy O. Laumann, Abraham Z. Snyder, Bradley L. Schlaggar, and Steven E. Petersen. 2014. “Methods to Detect, Characterize, and Remove Motion Artifact in Resting State fMRI.” NeuroImage 84 (Supplement C): 320–41. https://doi.org/10.1016/j.neuroimage.2013.08.048.

Pruim, Raimon H. R., Maarten Mennes, Daan van Rooij, Alberto Llera, Jan K. Buitelaar, and Christian F. Beckmann. 2015. “ICA-AROMA: A Robust ICA-Based Strategy for Removing Motion Artifacts from fMRI Data.” NeuroImage 112 (Supplement C): 267–77. https://doi.org/10.1016/j.neuroimage.2015.02.064.

Satterthwaite, Theodore D., Mark A. Elliott, Raphael T. Gerraty, Kosha Ruparel, James Loughead, Monica E. Calkins, Simon B. Eickhoff, et al. 2013. “An improved framework for confound regression and filtering for control of motion artifact in the preprocessing of resting-state functional connectivity data.” NeuroImage 64 (1): 240–56. https://doi.org/10.1016/j.neuroimage.2012.08.052.

Treiber, Jeffrey Mark, Nathan S. White, Tyler Christian Steed, Hauke Bartsch, Dominic Holland, Nikdokht Farid, Carrie R. McDonald, Bob S. Carter, Anders Martin Dale, and Clark C. Chen. 2016. “Characterization and Correction of Geometric Distortions in 814 Diffusion Weighted Images.” PLOS ONE 11 (3): e0152472. https://doi.org/10.1371/journal.pone.0152472.

Tustison, N. J., B. B. Avants, P. A. Cook, Y. Zheng, A. Egan, P. A. Yushkevich, and J. C. Gee. 2010. “N4ITK: Improved N3 Bias Correction.” IEEE Transactions on Medical Imaging 29 (6): 1310–20. https://doi.org/10.1109/TMI.2010.2046908.

Wang, Sijia, Daniel J. Peterson, J. C. Gatenby, Wenbin Li, Thomas J. Grabowski, and Tara M. Madhyastha. 2017. “Evaluation of Field Map and Nonlinear Registration Methods for Correction of Susceptibility Artifacts in Diffusion MRI.” Frontiers in Neuroinformatics 11. https://doi.org/10.3389/fninf.2017.00017.

Xu, J., Moeller, S., Auerbach, E. J., Strupp, J., Smith, S. M., Feinberg, D. A., Yacoub, E., Uğurbil, K. (2013). Evaluation of slice accelerations using multiband echo planar imaging at 3T. NeuroImage, 83, 991–1001.

Zhang, Y., M. Brady, and S. Smith. 2001. “Segmentation of Brain MR Images Through a Hidden Markov Random Field Model and the Expectation-Maximization Algorithm.” IEEE Transactions on Medical Imaging 20 (1): 45–57. https://doi.org/10.1109/42.906424.
